# Combining angiotensin receptor blockers with chlorthalidone or hydrochlorothiazide – which is the better alternative? A meta-analysis

**DOI:** 10.1186/s13643-020-01457-9

**Published:** 2020-08-24

**Authors:** Elena Filipova, Stela Dineva, Katya Uzunova, Velichka Pavlova, Krassimir Kalinov, Toni Vekov

**Affiliations:** 1 Science Department, Tchaikapharma High Quality Medicines, Inc, 1 G.M. Dimitrov Boulevard, 1172 Sofia, Bulgaria; 2 Department of Informatics, New Bulgarian University, 21 Montevideo Street, 1618 Sofia, Bulgaria; 3 Department of Pharmacy, Medical University, Pleven, Bulgaria

**Keywords:** Hydrochlorothiazide, Chlorthalidone, Diuretics, Angiotensin receptor blocker, Hypertension

## Abstract

**Background:**

Hypertension is a disease with significant clinical and socio-economic consequences. The reduction in cardiovascular mortality and morbidity in patients treated for hypertension is directly related to the magnitude of blood pressure reduction. Diuretics have proven useful for the prevention of cardiovascular complications in addition to a long history of safety and efficacy. The main aim for this meta-analysis is to compare the efficacy of the combination of angiotensin receptor blocker (ARB) and chlorthalidone (CTLD) to the combination of ARB and hydrochlorothiazide (HCTZ) in patients with hypertension.

**Methods:**

A comprehensive literature search was conducted through electronic databases PubMed, MEDLINE, Scopus, PsyInfo, Cochrane, eLIBRARY.ru, http://ClinicalTrials.gov and http://www.clinicaltrialsregister.eu in July 2020 to identify studies that investigate the effect of the combination of angiotensin receptor blocker with chlorthalidone or hydrochlorothiazide on the systolic and diastolic blood pressure in patients with hypertension. Changes in systolic and diastolic blood pressure (BP) expressed as a weighted mean difference (WMD) were our primary outcomes. The random-effects method was chosen as the primary analysis and results were presented with a 95% confidence interval (CI). Sensitivity analysis was performed and bias was assessed.

**Results:**

Our search returned 2745 titles. Of them, 51 full-text articles remained to be subjected to assessment. Comparisons of ARB/HCTZ versus ARB showed changes in BP of −6.89 (−8.09, −5.69) mmHg for systolic BP and − 3.67 (−4.15, −3.19) mmHg for diastolic BP. For the ARB/CTLD versus ARB/HCTZ comparison changes were − 6.30 (−7.30, −5.29) mmHg for systolic BP and − 3.57 (−4.17, 2.98) mmHg for diastolic BP.

**Conclusion:**

Our analysis suggests a small but significant favor for CTLD in blood pressure control when compared to HCTZ. We believe it should be considered as a valuable alternative for HCTZ and an option for fixed dose combinations with an ARB although further research is required.

## Background

Hypertension is a major global public-health challenge affecting approximately 1 billion individuals worldwide, with a projection to increase to 1.56 billion by 2025 given the increasingly aging population [1]. Hypertension is a disease with significant clinical and socio-economic consequences. Globally, cardiovascular diseases account for approximately 17 million deaths per year. More than a half of these cases are due to complications resulting from hypertension [2, 3]. The seventh report of the Joint National Committee on prevention, detection, evaluation, and treatment of high blood pressure describes the relationship between BP and risk of cardiovascular disease (CVD) as “continuous, consistent, and independent of other risk factors” [4]. The chances of heart attack, heart failure, stroke, and kidney diseases increase with BP increments. Meta-analysis of observational studies showed that the risk of cardiovascular death increases continuously from BP levels of 115 mmHg systolic and 75 mmHg diastolic [5].

The reduction in cardiovascular mortality and morbidity in patients treated for hypertension is directly related to the magnitude of blood pressure reduction. The utility of diuretics in the prevention of cardiovascular complications has been shown in major clinical trials [4], and these agents have a long history of safety and efficacy [6, 7]. Thiazide-like/type diuretics, such as chlorthalidone (CTLD) and hydrochlorothiazide (HCTZ), respectively, are important options for use in uncomplicated hypertension, without the presence of comorbid conditions. There has been debate about whether thiazide-like diuretics such as chlorthalidone and indapamide should be given preference over classical thiazide diuretics (e.g., hydrochlorothiazide and bendrofluazide), but their superiority on outcomes has never been tested in head-to-head RCTs [8]. For instance, CTLD is 1.5 to 2.0 times more potent than HCTZ on an mg:mg basis, and has a considerably longer half-life (45–60 h vs 8–15 h) and duration of action (48–72 h vs 16–24 h) after long-term dosing. Meta-analyses also suggest that CTLD is superior to HCTZ in preventing cardiovascular events [9, 10]. A meta-analysis [11] and a network meta-analysis (being prepared for publication) performed by our team also point to a prevalence for CTLD with regard to systolic and diastolic blood pressure control.

However, many treated hypertensive patients have inadequate blood pressure control and do not attain treatment goal [12, 13]. Although a single antihypertensive agent is considered to be the ideal in terms of convenience and compliance, many patients with essential hypertension require a combination drug regimen [14, 15].

Combination therapy with a renin-angiotensin system (RAS) inhibitor (either an angiotensin-converting enzyme inhibitor or an angiotensin II receptor blocker [ARB]) plus a diuretic is a widely used and effective approach that has become an established component of evidence-based hypertension treatment guidelines [16–18]. The combination of an ARB with a thiazide diuretic has been shown to be efficacious and well tolerated in numerous clinical trials [19–29]. This combination could be of particular value in hypertensive patients with additional cardiovascular risk factors or in populations whose BP is traditionally poorly controlled, such as elderly persons, persons with diabetes mellitus and black patients [19, 30]. However, the question of which diuretic—chlorthalidone or hydrochlorothiazide has not been widely discussed.

## Materials and methods

### Main aim

The main aim for this meta-analysis is to compare the efficacy of the combination of angiotensin receptor blocker and chlorthalidone to the effect of the combined use of angiotensin receptor blocker and hydrochlorothiazide in adult patients with hypertension.

### Data sources and search strategy

A comprehensive literature search was conducted through electronic databases: Cochrane, eLIBRARY.ru, MEDLINE, PsyInfo, PubMed, Scopus, and registries for data of clinical trials (http://ClinicalTrials.gov and http://www.clinicaltrialsregister.eu) in September 2018 to identify studies that investigate the effect of the combination of angiotensin receptor blocker with chlorthalidone or hydrochlorothiazide on the systolic and diastolic blood pressure in patients with hypertension. The following keywords and various combinations were used in the search: *hydrochlorothiazide*, *chlorthalidone*, *diuretics*, *ARB*, *angiotensin receptor blocker*, *hypertension*, *blood pressure*, *clinical trial*, *controlled*, randomi*, *double blind.* Results were not limited only to those in English. Similar key words and their combinations were used in Cyrillic: *гидрохлортиазид*, *хлорталидон*, *диуретики*, *AR блокеры*, *ARB*, *повышеное давление*, *артериальная гипертензия*, *кровяное давление*, *артериальное давление*, *клиническое испытание*, *клиническое исследование*, *контролированное*, *рандомизированное*, *двойное слепое*. Unfortunately, results in Cyrillic were not found. Full-text articles and abstracts were checked for relevance to the topic and were assessed. Generally, we did not restrict the search period but relevant articles were mainly published in the 1980-2020 period.

### Inclusion criteria

Search results were assessed for relevance on the basis of the following inclusion criteria: (1) type of study/trial—epidemiologic, controlled, and randomized; (2) investigation of the effect the combination of angiotensin receptor blocker with chlorthalidone or hydrochlorothiazide on the systolic and diastolic blood pressure; (3) type of subjects included—representatives of the whole population or a specific stratum; (4) patients with essential hypertension; (5) access to raw data; (6) eligibility for statistical analysis. If any clarification of results or conclusions was needed, authors were contacted for additional information. We have not limited our search to a particular angiotensin receptor blocker but have attempted to review the group as a whole. Sources were excluded if they represented trials in which the principle arm reported other outcomes different from changed in systolic and diastolic blood pressure; other conditions apart from hypertension; other combinations of CTLD and HCTZ (for example with calcium channel blockers and angiotensin converting enzyme inhibitors).

### Quality assessment

Effective public health practice project was utilized to assess study quality. This tool includes assessment of different characteristics like selection bias, study design, blinding, data collection method, confounders, and drop outs in order to help raters form an opinion of quality based upon information contained in the study. Studies that correspond to the aforementioned inclusion criteria are subjected to a quality estimation and general ratings are taken into account when results from the study are interpreted. Overall quality of evidence for the two primary outcomes was assessed according to Grade methodology.

### Data extraction and statistical analysis

**Fig. 1:**
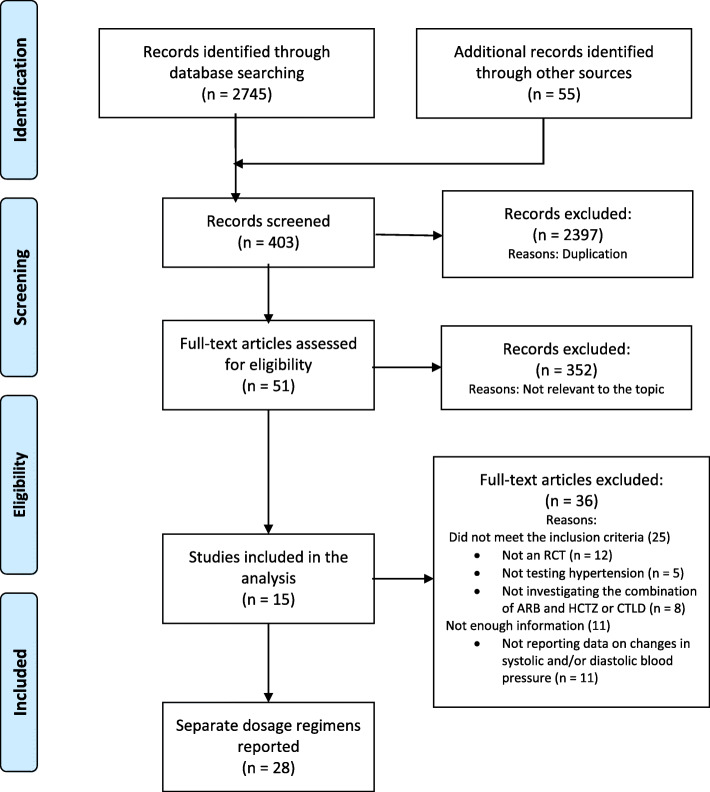
Flow chart of study selection process

All relevant studies identified were carefully reviewed, sorted, and assessed. Figure 1 depicts the process of selection applied to evaluated studies in order to determine their eligibility for inclusion in the analysis. Extracted data encompassed publication year, type of study, ARB used, type of population, duration of study, number of patients, doses used of the ARB and the diuretic. Additionally, data about measurements of systolic and diastolic blood pressure were extracted separately in Excel. Data for systolic and diastolic blood pressure was presented as weighed mean difference with a 95% confidence interval (CI).

Because of the significant heterogeneity of the individual studies, we chose the random-effects method as the primary analysis. To assess the aforementioned heterogeneity of treatment effect among trials, we used the Cochran *Q* and the *I*2 statistics, where *p* values of less than 0.10 were used as an indication of the presence of heterogeneity and an *I*2 parameter greater than 50% was considered indicative of substantial heterogeneity [31]. The threshold for statistical significance was set at 0.05. Forest plots depict estimated results from the studies included in the analysis. Funnel plots were used to evaluate publication bias (not shown in the main manuscript, but provided as Supplementary material). Sensitivity analyses were performed in order to evaluate the degree of influence of the consequent elimination of each individual study on the final result. Calculations were made with MetaXL ver 5.3 (add-ins of MSExel).

## Results

Our search returned 2745 titles. Once duplicate reports and studies not relevant to the analysis were excluded, 51 full text articles remained to be subjected to assessment. After evaluation based on the inclusion criteria described in the methods, only 15 studies presenting data for 28 separate dose regimens remained to be included in the analysis comparing the efficacy of the combination of angiotensin receptor blocker and chlorthalidone to the effect of the combined use of angiotensin receptor blocker and hydrochlorothiazide in patients with hypertension (Fig. 1).

Characteristics of the studies included in the meta-analysis are outlined in Table 1 and data about baseline systolic and diastolic blood pressure is presented in Table 2. All 15 studies [20–29, 32–36] are randomized and all but 3 [23, 27, 36] are double blinded. There are 10 studies comparing ARB/HCTZ with HCTZ [20–22, 24–29, 32], one study comparing ARB/CTLD with CTLD [33] and 4 studies comparing ARB/CTLD with ARB/HCTZ [23, 34, 36]. In 4 studies, the ARB used is olmesartan [20, 34–36], in 3 [21, 26, 27] it is valsartan, in 3 [22, 23, 27] it is candesartan, and in 3 it is telmisartan [24, 25, 27]. There is one study discussing each of the following: fimasartan [28], eprosartan [29], and losartan [32]. Azilsartan is used for treatment in 4 studies [33–36]. Most commonly studies last 8 weeks although Cushman et al. [34] and Makita et al. [27] use duration of 12 weeks, Fogari et al. [20]—16 weeks, and Neutel et al. [36]—52 weeks. The number of patients involved varies a lot between studies but as a whole 2515 patients are treated with angiotensin receptor blocker, 4095 are treated with the combination of ARB/HCTZ, and 2795 are treated with the combination ARB/CTLD. The quality of the studies has been estimated as poor, moderate, or strong using the effective public health practice project. Results are presented in Table S1 the Supplementary material.

**Table 1:** Study characteristics

Author, year	Type of study	ARB used	Type of population	Duration of study	Number of patients			Doses, mg		
					ARB	HCTZ + ARB	CTDN + ARB	ARB	HCTZ + ARB	CTDN + ARB
Fogari et al., 2010 [20]	Randomized, double-blind, parallel-group, up-titration, multicenter, multinational, phase III	Olmesartan	Moderate to severe hypertension, male or female patients, mean age 55.6	16 weeks	285	556		40	40/12.5, 40/25	
Benz et al., 1998 [21]	Randomized, double-blind, multiple dose, placebo controlled, multifactorial, parallel	Valsartan	Uncomplicated essential hypertension, male or female patients, mean age 52 (22-86)	8 weeks	198 (99, 99)	379 (96, 92, 97, 94)		80, 160	80/12.5, 80/25, 160/12.5, 160/25	
Edes, 2009 [22]	Randomized, double-blind, parallel-group study	Candesartan	Mild to moderate primary hypertension, male or female patients, mean age 53	8 weeks, 4 weeks follow-up	465	492		32	32/25	
Kwon et al., 2013 [23]	Open-label, randomized, prospective cross-over study	Candesartan	Never treated primary hypertension, male or female patients, mean age 50	8 weeks	25 (4 weeks)	15	13	8	8/25	8/12.5
Lacourciere and Martin, 2002 [24]	Prospective, randomized, double-blind, parallel-group study	Telmisartan	Mild-to-moderate essential hypertension, male or female patients, mean age 54.1 (28-79)	8 weeks	167	160		40	40/12.5	
Lacourciere et al., 2001 [25]	Multicenter, prospective, randomized, double-blind, parallel-group study	Telmisartan	Mild-to-moderate, essential hypertension and inadequate BP control, male or female patients, mean age 55.6 (20-79)	8 weeks	245	246		80	80/12.5	
Lacourciere et al., 2005 [26]	Randomized, double blind, 3-arm, parallel group study	Valsartan	Stage 2 or 3 systolic hypertension (SBP ≥ 160 mmHg and ≤ 200 mmHg) with or without other CV risk factors, male or female patients, mean age 60.8	8 weeks	261	513 (258, 255)		80, 160	160/12.5, 160/25	
Makita et al., 2009 [27]	Randomized, parallel-group study	Candesartan/valsartan vs telmisartan	Hypertensive outpatients treated with an ARB, candesartan or valsartan, male or female patients, mean age 69.3	12 weeks	32	32		8 or 80	40/12.5	
Rhee et al., 2015 [28]	Multicenter, randomized, active-controlled, double-blind, parallel-group, dose-titration trial	Fimasartan	Mild to moderate primary hypertension, male or female patients, mean age 55.3	8 weeks	88	175		60	60/12.5	
Sachse et al., 2002 [29]	Multicenter, prospective, randomized, double-blind, parallel group study	Eprosartan	Mild to moderate primary hypertension, male or female patients mean age 58.7	8 weeks, 4 weeks follow-up	157	152		600	600/12.5	
MacKay et al., 1996 [32]	Multicenter, randomized, double-blind, parallel-group study	Losartan	Essential hypertension, male or female patients, mean age 55 (22-79)	8 weeks, 4 weeks follow-up	122	125, 114		50	50/6.25, 50/12.5	
Sica et al., 2012 [33]	Phase 3, randomized, double-blind, factorial study	Azilsartan	Mild to moderate primary hypertension, male or female patients, mean age 57	8 weeks	470 (155, 153, 162)		928 (156, 147, 153, 154, 156, 162)	20, 40, 80		20/12.5, 40/12.5, 80/12.5, 20/25, 40/25, 80/25
Cushman et al., 2012 [34]	Randomized, double-blind, forced-titration study	Azilsartan+ chlorthalidone; olmesartan+ hydrochlorothiazide	Primary hypertension, male or female patients, mean age 57	12 weeks		364	355, 352		40/25	40/25, 80/25
Cushman et al., 2018 [35]	Randomized, double-blind, parallel-group study	Azilsartan+ chlorthalidone; olmesartan+ hydrochlorothiazide	Primary hypertension, male or female patients, mean age 57	8 weeks		356	372, 357		40/25	40/25, 80/25
Neutel et al., 2017 [36]	Phase 3, randomized, parallel-group, open-label, multicenter, multinational study	Azilsartan+ chlorthalidone; olmesartan+ hydrochlorothiazide	Stage 2 essential hypertension, male or female patients, mean age 58.5 (> 45)	52 weeks		419	418		40/25	80/25

Note: Mild to moderate hypertension stands for stage I and stage II hypertension according to “2017 High Blood Pressure Clinical Practice Guideline”

Normal: Systolic < 120 and diastolic < 80 mmHg

Elevated: Systolic between 120 and 129 and diastolic < 80

Stage 1: Systolic between 130 and 139 or diastolic between 80 and 89

Stage 2: Systolic ≥ 140 or diastolic ≥ 90 mmHg

**Table 2:** Baseline systolic and diastolic blood pressure

Author, year	SBP			DBP		
	ARB	HCTZ + ARB	CTDN + ARB	ARB	HCTZ + ARB	CTDN + ARB
Fogari et al., 2010 [20]	168.1 ± 7.6	168.5 ± 8.4		104.5 ± 4.0	104.6 ± 4.2	
Benz et al, 1998 [21]	153.7 ± 14.4 153.5 ± 15.1	153.0 ± 14.0154.5 ± 15.4152.0 ± 14.2155.9 ± 14.8		101.5 ± 4.9 101.5 ± 4.8	101.0 ± 4.9 101.0 ± 4.5 100.4 ± 4.6 101.4 ± 4.8	
Edes, 2009 [22]	152.9 ± 12.8	154.0 ± 13.1		97.4 ± 5.6	97.5 ± 5.6	
Kwon et al., 2013 [23]	153 ± 13	128 ± 14	131 ± 12	94 ± 8	81 ± 11	84 ± 9
Lacourciere and Martin, 2002 [24]	146.7 ± 12.7	147.1 ± 13.6		95.6 ± 4.8	95.7 ± 4.7	
Lacourciere et al., 2001 [25]	148.7 ± 16.1	148.9 ± 14.8		96.6 ± 5.2	96.4 ± 6.0	
Lacourciere et al., 2005 [26]	167.9 ± 8.0	167.4 ± 8.3 167.2 ± 7.9		93.2 ± 8.9	93.4 ± 9.6 93.7 ± 8.8	
Makita et al., 2009 [27]	160.6 ± 10.9	162.5 ± 10.9		84.5 ± 7.8	86.1 ± 8.7	
Rhee et al., 2015 [28]	150.8 ± 12.7	149.4 ± 11.9		96.8 ± 5.7	96.5 ± 5.428	
Sachse et al., 2002 [29]	156.0 ± 1.1	155.3 ± 1.1		98.9 ± 0.4	99.9 ± 0.4	
MacKay et al., 1996 [32]	152.2	152.6 151.3		100.9	101.2 101.7	
Sica et al, 2012 [33]	163 164 164		165 165 165 165 164 164	95 95 95		95 96 94 96 94 94
Cushman et al, 2012 [34]		164.7 ± 9.9	164.9 ± 10.1 164.8 ± 9.8		95.2 ± 10.3	96.1 ± 9.8 95.9 ± 9.8
Cushman et al, 2018 [35]		164.7 ± 10.4	165.2 ± 11.1 164.9 ± 10.5		96.1 ± 10.4	95.3 ± 10.5 95.4 ± 10.0
Neutel et al., 2017 [36]		167.6 ± 7.0	168.2 ± 7.1		95.7 ± 9.6	95.7 ± 9.2

### ARB/HCTZ versus ARB

**Fig. 2:**
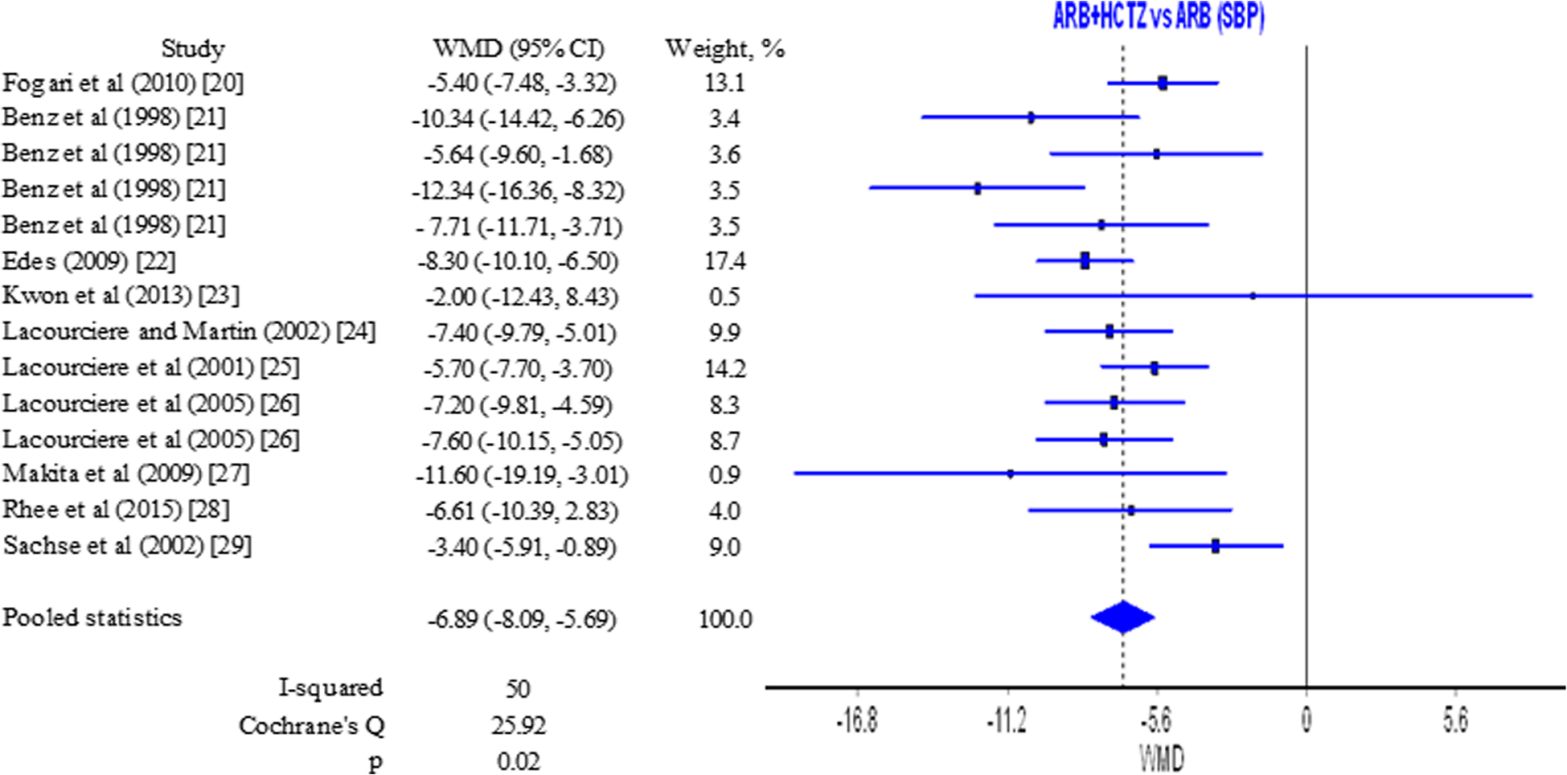
Forest plot for systolic blood pressure ARB/HCTZ versus ARB

Data from 10 studies [20–29] with 1923 patients treated with ARB alone and 2720 patients treated with the combination ARB/HCTZ was used to estimate the effect of therapies on systolic blood pressure. Pooled results showed a weighted mean difference (WMD) of −6.89 (−8.09, −5.69) mmHg (Fig. 2) which is statistically significant (*p* = 0.02). *I*^2^ and *Q* values signify a moderate heterogeneity of results. Bias has been assessed (Figure S1 in Supplementary material).

**Fig. 3:**
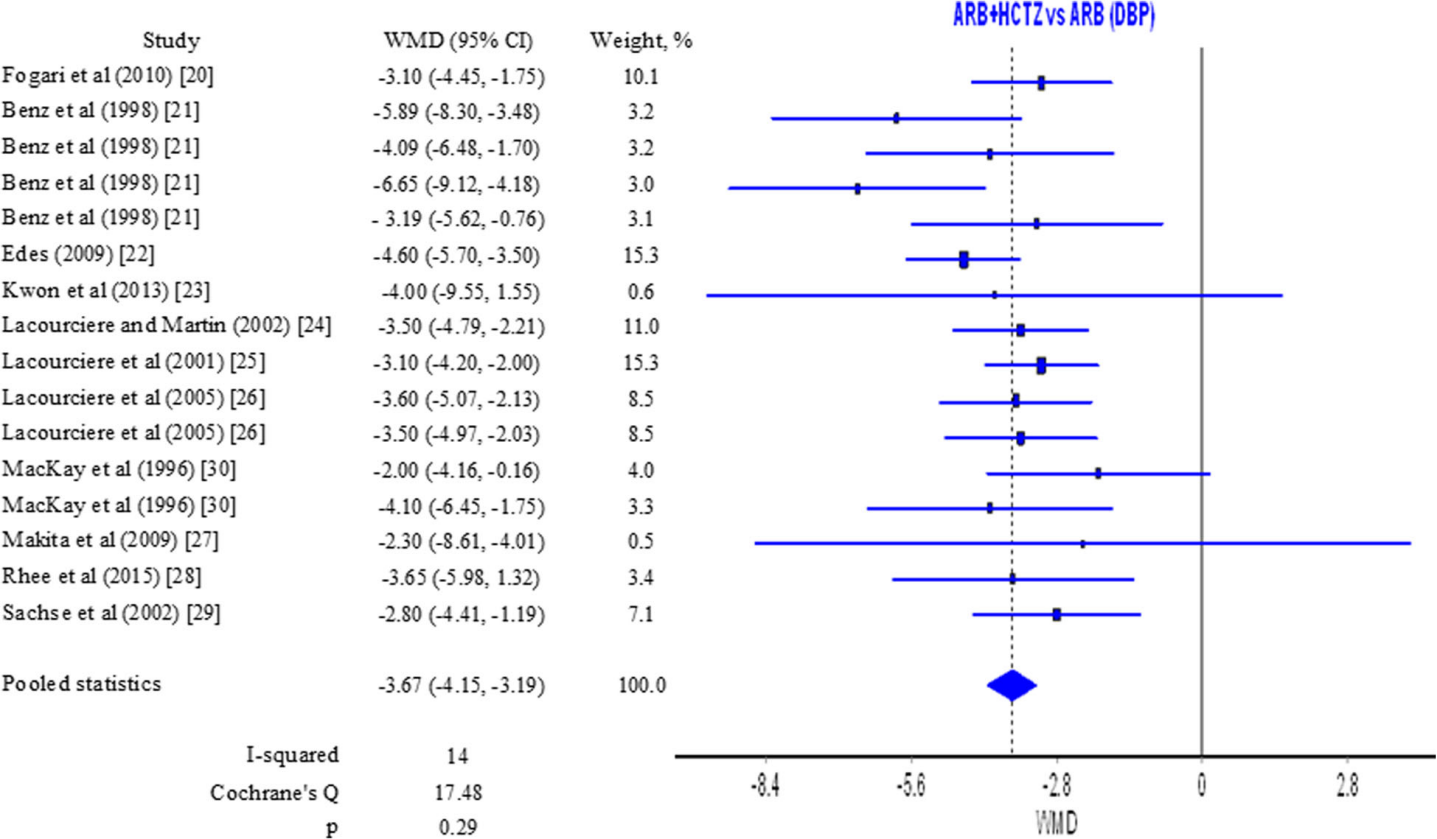
Forest plot for diastolic blood pressure ARB/HCTZ versus ARB

Effect of ARB alone versus ARB/HCTZ on diastolic blood pressure was estimated on the basis of results from 11 studies [20–29, 32]. Pooled results showed a WMD of −3.67 (−4.15, −3.19) mmHg (Fig. 3). The difference is in favor of the combination of ARB/HCTZ but it is statistically insignificant. *I*^2^ and *Q* values in this case are a marker for homogeneity and coherence among results (Fig. 3 and Figure S2 in Supplementary material). Reductions in systolic and diastolic blood pressure can be considered clinically significant due to considerable reduction in the risk for cardiovascular complications [37, 38].

### ARB/CTLD versus ARB

Only one article was found that compared the effect of the combination of ARB/CTLD on the systolic blood pressure to the effect of monotherapy with ARB. Sica et al. compared treatment with azilsartan medoxomil (AZL-M) 20 mg, 40 mg, or 80 mg; CTLD 12.5 or 25 mg; or 1 of the 6 combinations of these doses (AZL-M/CTLD: 20/12.5 mg, 40/12.5 mg, 80/12.5 mg, 20/25 mg, 40/25 mg, and 80/25 mg). Their primary endpoint was the change in systolic blood pressure determined by an ambulatory blood pressure measurement. The authors conclude that for the pooled AZL-M/CLD 40/25-mg and 80/25-mg FDC groups, SBP reduction by ABPM was 28.9 mmHg and exceeded AZL-M 80 mg and CLD 25 mg monotherapies by 13.8 mmHg and 13 mmHg, respectively (*p* < .001 for both comparisons). They also comment that the incremental reduction in blood pressure for the combination containing 25 mg CTLD is significantly greater than what has previously been seen with an FDC containing 25 mg of HCTZ [33].

### ARB/CTLD versus ARB/HCTZ

**Fig. 4:**
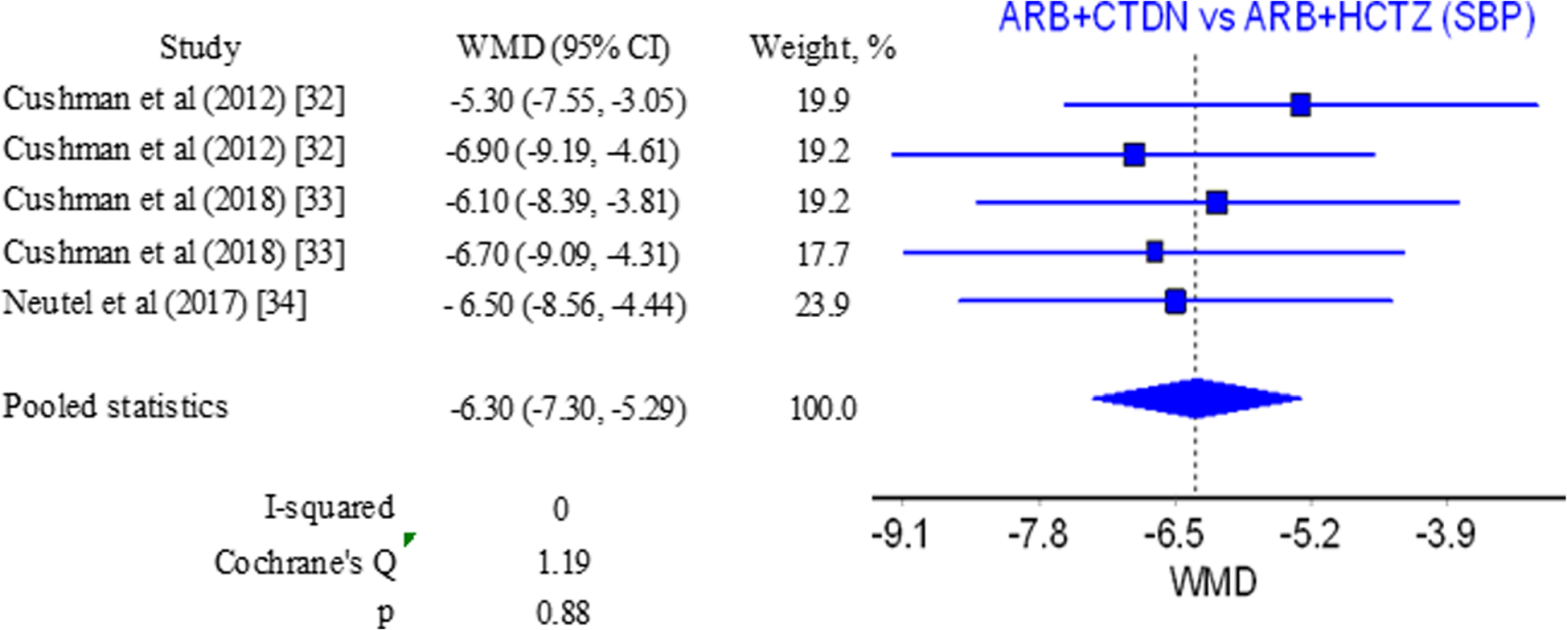
Forest plot for systolic blood pressure ARB/CTLD vs ARB/HCTZ

Finally, we attempted to compare the effect of the two combinations on systolic and diastolic blood pressure in patients with hypertension. Only 3 studies were found that report results concerning changes in systolic blood pressure [34–36]. It should be noted that all of them are fairly recent, pointing out to a renewed interest in the potential of CTLD. Pooled results show a WMD of −6.30 mmHg (−7.30, −5.29) in favor of the combination containing CTLD (Fig. 4). *I*^2^ and *Q* values in this case are a marker for homogeneity and coherence among results (Figure S3 in Supplementary material).

**Fig. 5:**
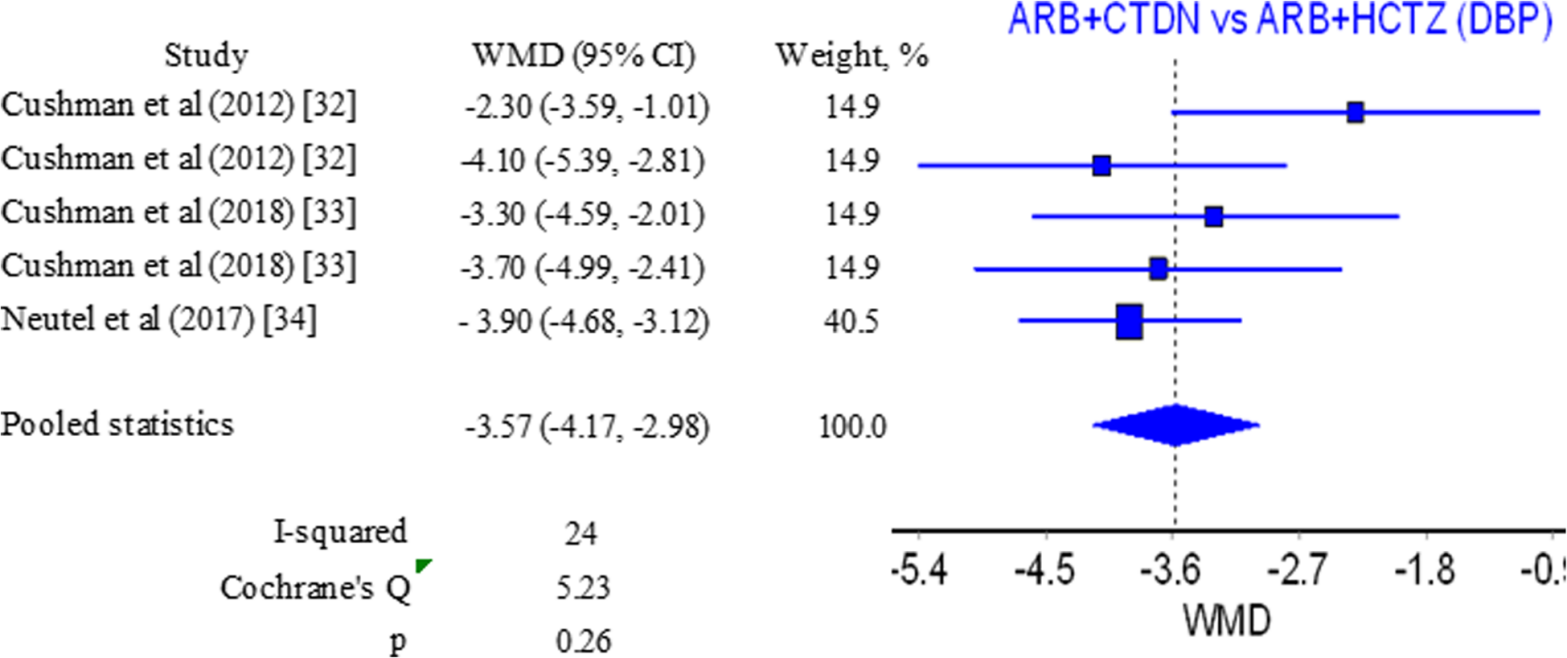
Forest plot for diastolic blood pressure ARB/CTLD vs ARB/HCTZ

Same 3 studies report results concerning diastolic blood pressure [34–36]. Pooled WMD in this case is −3.57 mmHg (−4.17, 2.98) (Fig. 5) with prevalence for ARB/CTLD combination. *I*^2^ and *Q* values in this case are a marker for homogeneity and coherence among results (Figure S4 in Supplementary material). Both values for WMD in this case are statistically insignificant but can be considered clinically significant due to considerable reduction in the risk for cardiovascular complications [37, 38].

### Sensitivity analysis

Results from sensitivity analyses in relation to systolic and diastolic blood pressure for the comparisons ARB/HCTZ vs ARB and ARB/CTLD vs ARB/HCTZ respectively are presented in Tables S2 and S3 in the Supplementary material. When each study was subsequently excluded from the analysis, pooled WMD for systolic blood pressure for the ARB/HCTZ vs ARB comparison was in the range −7.23 to −6.59 mmHg while for diastolic blood pressure for the same comparison the range was −3.77 to −3.50 mmHg. Although studies have varying weights (Figs. 2 and 3), the subsequent exclusion of each study does not lead to significant change in results. The lack of substantial changes in WMD suggests consistency in findings and is a tentative confirmation of the possible prevalence for the combination compared to the monotherapy.

Pooled WMD for systolic blood pressure for the ARB/CTLD vs ARB/HCTZ comparison is presented in Table S3 in the Supplementary material and is in the range −6.54 to −6.15 mmHg. For the diastolic blood pressure, these values are in the range −3.80 to −3.35 mmHg. Studies have similar weights (Figs. 4 and 5) and the variation in the values with subsequent exclusion of studies is very small. The results confirm the prevalence for the combination of ARB and CTLD.

## Discussion

The combination of ARB and thiazide diuretics is highly effective for the treatment of hypertension and is well tolerated at the same time [39, 40]. This combination can be given as initial therapy (where appropriate) or later in the course of treatment [4]. Most commonly, the ARB is combined with hydrochlorothiazide despite the existence of various diuretics [40]. Recent studies suggest that chlorthalidone may be a suitable if not better alternative for hydrochlorothiazide [34–36, 41]. NICE guidelines for the diagnosis and treatment of hypertension recommend ARB or angiotensin converting enzyme inhibitors as a starting regimen and advise the addition of thiazide-like diuretics as CTLD as opposed to hydrochlorothiazide [42]. However, elderly patients with hypertension often have other comorbidities which require additional medications. Polypharmacy is associated with increased risk of adverse events (fall injury, hyperkalemia and hypokalemia, heart failure, and blood pressure exacerbation), polypharmacy mismanagement, drug-drug interaction, and increased costs [43]. For such patients, treatment should be individualized and innovative approaches such as use of a fixed-dose combination pill, ingestible sensor system, electronic reminder system, medical audits, and the integration of a pharmacist in the care of patients should be implemented to avoid polypharmacy mismanagement [43]. A retrospective observational medical chart review study suggests that adopting the clinical pharmacist’s recommendations in a collaborative care approach could reduce the number of prescribed potentially inappropriate medications in patients aged 65 years or more therefore reducing the harmful drug-drug interactions and improving the adherence to treatment guidelines [44]. Another study also suggests that a collaborative approach to address the risks of drug–drug interactions and that the potential use of drug–drug interaction checkers could be beneficial [45].

Considering all said above, optimal control of blood pressure in hypertensive patients should be the goal. Although that may not always be achievable results of meta-analysis which used data from 147 randomized clinical trials showed that blood pressure reduction of 10 mmHg systolic and 5 mmHg diastolic was associated with a 41% (33 to 48%) reduction in stroke for all trials, 46% (35-55 %) in primary prevention trials, 44% (21-44 %) in secondary prevention trials, and 35% (20-47 %) in trials including subjects with a history of coronary heart disease [37]. Other authors claim that even reductions of −2 mmHg can be considered clinically significant [38, 46].

Our analysis suggest as many have before us [33, 47–52] that the combination of ARB and a thiazide diuretic is more effective for the control of systolic and diastolic blood pressure than the use of the ARB or diuretic alone. When it comes to a comparison between the combinations ARB/HCTZ and ARB/CTLD pooled results show a WMD of −6.30 mmHg (−7.30, −5.29) in favor of the combination containing CTLD for control of systolic blood pressure. A WMD of −3.57 mmHg (−4.17, 2.98) for control of diastolic blood pressure is in favor of CTLD again. Our results suggest a prevalence for CTLD over HCTZ when the two are combined with ARB and used for blood pressure reduction in patients with hypertension.

It should be noted that a certain degree of heterogeneity exists in the studies which were deemed eligible and were included in the analysis. This can be attributed to multiple factors such as variety of study design and outcomes; differences in the inclusion and exclusion criteria; the way of measuring BP; different ARBs used; and varying doses of diuretics. We have, however, attempted to determine the significance of those differences by performing sensitivity analysis.

Interest in CTLD was renewed of late. MRFIT is the first trial to suggest superiority of CTLD. Results from MRFIT show that replacement of HCTZ with CTLD might lead to lower coronary heart disease mortality [53]. Dorsch et al. conducted a retrospective cohort analysis of MRFIT and concluded that after a follow-up period of 6 years cardiovascular events were less frequent in the CTLD group—21% lower than with HCTZ (*p* = 0.0016) [54]. This estimation was confirmed by a network meta-analysis which declared that CTLD was better for preventing cardiovascular events in patients with hypertension reducing them by 21% [10]. At the same time another observational cohort study does not find any association between reduction of cardiovascular events and CTLD use while at same time it shows an increase in the cases of electrolyte disturbances, specifically hypokalemia [55]. We have also attempted to estimate the effect of the combination of CTLD and HCTZ with ARB on the serum levels of sodium and potassium. Only two studies [35, 36] reported data on levels of serum potassium. The WMD in this case was only 0.01 mEq/L suggesting lack of difference in the effect of the two combinations. Data for levels of serum sodium was unfortunately scarce.

There are several studies investigating the effect of CTLD and HCTZ on blood pressure. Greater reduction in SBP was recorded in a small randomized, single-blinded, crossover study comparing CTLD 12.5 mg/day (force-titrated to 25 mg/day) and HCTZ 25 mg/day (force-titrated to 50 mg/day) [56] and in a double-blind, double-dummy, randomized, parallel group, comparative, multicentric trial [57]. A meta-analysis comparing the dose response of HCTZ, CTLD, and bendroflumethiazide on blood pressure predicted that reduction of 10 mmHg of SBP could be achieved by 1.4, 8.6, and 26.4 mg of bendroflumethiazide, CTLD and HCTZ respectively [58].

We evaluated a large number of sources in our attempt to examine the interchangeability of HCTZ and CTLD when they are used in combination with an ARB by assessing their effect on systolic and diastolic blood pressure. We tried to draw conclusions only by referring to trials we deemed to be of satisfactory quality (see Supplementary material). Even though we used a lot of sources and included numerous patients in the analysis which is a prerequisite for reduction of bias we also used funnel plots in order to assess bias (see Supplementary material). Additionally, we did not manage to find another paper be it a meta-analysis or a systematic review focusing on our topic and comparing the efficacy of the combination of angiotensin receptor blocker (ARB) and chlorthalidone (CTLD) to the combination of ARB and hydrochlorothiazide (HCTZ) in patients with hypertension.

There are a number of limitations intrinsic to the analysis. First of all, high quality trials investigating the efficacy of CTLD combined with ARB are scarce. Secondly, we have evaluated the effects of HCTZ and CTLD using data for combined doses. Almost all studies included in our statistical analysis were conducted relatively recently and all but two were funded by industry. Additionally, we did not take into consideration any existing comorbidities of the patients.

## Conclusion

The combination of an ARB with a diuretic is a widely used method for addressing hypertension as a first or subsequent line of treatment. The most commonly used diuretic seems to be HCTZ. Our analysis suggests that CTLD should be considered as a valuable alternative for HCTZ and an option for fixed dose combinations with an ARB due to its tentative prevalence in blood pressure control when compared to HCTZ.

## Supplementary information

**Additional file 1: Table S1.** Quality assessment results. **Figure S1.** Funnel plot for systolic blood pressure ARB/HCTZ versus ARB. **Figure S2.** Funnel plot for diastolic blood pressure ARB/HCTZ versus ARB. **Figure S3.** Funnel plot for systolic blood pressure ARB/CTLD vs ARB/HCTZ. **Figure S4.** Funnel plot for diastolic blood pressure ARB/CTLD vs ARB/HCTZ. **Table S2.** Sensitivity analysis SBP and DBP ARB/HCTZ vs ARB. **Table S3.** Sensitivity analysis SBP and DBP ARB/CTLD vs ARB/HCTZ. **Table S4.** Quality of the evidence according to the GRADE methodology for the 2 outcomes.

## Data Availability

All data generated or analyzed during this study are included in this published article and its supplementary information files.

## Abbreviations

BP: Blood pressure
CVD: Cardiovascular disease
CTLD: Chlorthalidone
HCTZ: Hydrochlorothiazide
RAS: Renin-angiotensin system
ARB: Angiotensin II receptor blocker
CI: Confidence interval
WMD: Weighed mean difference
AZL: Azilsartan

## References

[1] Kearney PM, Whelton M, Reynolds K, Muntner P, Whelton PK, He J (2005). Global burden of hypertension: analysis of worldwide data. Lancet.

[2] A global brief on hypertension: silent killer, global public health crisis: World Health Day (2013). World Health Organization;2013.

[3] Petrov A, Gatchev E, Kalinov K, Filipova E, Uzunova K, Vekov Y (2018). Comparative bioavailability of a newly developed irbesartan 300 mg containing preparation. Hosp Pharm.

[4] Chobanian AV, Bakris GL, Black HR, Cushman WC, Green LA, Izzo JL (2003). Seventh report of the joint National Committee on prevention, detection, evaluation, and treatment of high blood pressure. Hypertension.

[5] Lewington S, Clarke R, Qizilbash N, Peto R, Collins R (2002). Age-specific relevance of usual blood pressure to vascular mortality. Lancet..

[6] ALLHAT Officers and Coordinators for the ALLHAT Collaborative Research Group (2002). Major outcomes in high-risk hypertensive patients randomized to angiotensin-converting enzyme inhibitor or calcium channel blocker vs diuretic: the antihypertensive and lipid-lowering treatment to prevent heart attack trial (ALLHAT). JAMA..

[7] Psaty BM, Lumley T, Furberg CD, Schellenbaum G, Pahor M, Alderman (2003). Health outcomes associated with various antihypertensive therapies used as first-line agents. A network meta-analysis. JAMA.

[8] Roush GC, Ernst ME, Kostis JB, Tandon S, Sica DA (2015). Head-to-head comparisons of hydrochlorothiazide with indapamide and chlorthalidone: antihypertensive and metabolic effects. Hypertension.

[9] Roush GC, Buddharaju V, Ernst ME (2013). Is chlorthalidone better than hydrochlorothiazide in reducing cardiovascular events in hypertensives?. Curr Opin Cardiol.

[10] Roush GC, Holford TR, Guddati AK (2012). Chlorthalidone compared with hydrochlorothiazide in reducing cardiovascular events: systematic review and network meta-analyses. Hypertension..

[11] Dineva S, Uzunova K, Pavlova V, Filipova E, Kalinov K, Vekov T (2019). Comparative efficacy and safety of chlorthalidone and hydrochlorothiazide-meta-analysis. J Hum Hypertens.

[12] Guidelines Subcommittee (1999). 1999 World Health Organization-International Society of Hypertension guidelines for the management of hypertension. J Hypertens.

[13] Mancia G, De Backer G, Dominiczak A, Cifkova R, Fagard R, Germano G (2007). 2007 guidelines for the management of arterial hypertension: the task force for the management of arterial hypertension of the European Society of Hypertension (ESH) and of the European Society of Cardiology (ESC). J Hypertens.

[14] Joint National Committee on Detection, Evaluation and Treatment of High Blood Pressure (1993). The fifth report of the joint National Committee on detection, evaluation and treatment of high blood pressure (JNCV). Arch Intern Med.

[15] Epstein M, Bakris G (1996). Newer approaches to antihypertensive therapy. Use of fixed dose combination therapy. Arch Intern Med.

[16] James PA, Oparil S, Carter BL, Cushman WC, Dennison-Himmelfarb C, Handler J (2014). 2014 evidence-based guideline for the management of high blood pressure in adults: report from the panel members appointed to the eighth joint National Committee (JNC 8). JAMA..

[17] Weir MR, Bakris GL (2008). Combination therapy with renin-angiotensin-aldosterone receptor blockers for hypertension: how far have we come?. J Clin Hypertens (Greenwich).

[18] Sood N, Reinhart KM, Baker WL (2010). Combination therapy for the management of hypertension: a review of the evidence. Am J Health Syst Pharm.

[19] Mimran A, Weir MR (2005). Angiotensin-receptor blockers and diuretics--advantages of combination. Blood Press.

[20] Fogari R, Taddei S, Holm-Bentzen M, Baszak J, Melani L, Schumacher K (2010). Efficacy and safety of olmesartan medoxomil 40 mg/hydrochlorothiazide 12.5 mg combination therapy versus olmesartan medoxomil 40 mg monotherapy in patients with moderate to severe hypertension: a randomized, double-blind, parallel-group, multicentre, mult. Clin. Drug Investig.

[21] Benz JR, Black HR, Graff A, Reed A, Fitzsimmons S, Shi Y (1998). Valsartan and hydrochlorothiazide in patients with essential hypertension. A multiple dose, double-blind, placebo controlled trial comparing combination therapy with monotherapy. J Hum Hypertens.

[22] Edes I, Group MS (2009). Combination therapy with candesartan cilexetil 32 mg and hydrochlorothiazide 25 mg provides the full additive antihypertensive effect of the components: a randomized, double-blind, parallel-group study in primary care. Clin Drug Investig.

[23] Kwon BJ, Jang SW, Choi KY, Kim DB, Cho EJ, Ihm SH (2013). Comparison of the efficacy between hydrochlorothiazide and chlorthalidone on central aortic pressure when added on to candesartan in treatment-naïve patients of hypertension. Hypertens Res.

[24] Lacourciere Y, Martin K (2002). Comparison of a fixed-dose combination of 40 mg telmisartan plus 12.5 mg hydrochlorothiazide with 40 mg telmisartan in the control of mild to moderate hypertension. Am J Ther.

[25] Lacourcière Y, Tytus R, O’Keefe D, Lenis J, Orchard R, Martin K (2001). Efficacy and tolerability of a fixed-dose combination of telmisartan plus hydrochlorothiazide in patients uncontrolled with telmisartan monotherapy. J Hum Hypertens.

[26] Lacourcière Y, Poirier L, Hebert D, Assouline L, Stolt P, Rehel B (2005). Antihypertensive efficacy and tolerability of two fixed-dose combinations of valsartan and hydrochlorothiazide compared with valsartan monotherapy in patients with stage 2 or 3 systolic hypertension: an 8-week, randomized, double-blind, parallel-group T. Clin Ther.

[27] Makita S, Abiko A, Naganuma Y, Tamada M, Nakamura M (2009). Efficacy of low-dose hydrochlorothiazide in combination with telmisartan on early morning blood pressure in uncontrolled hypertensive patients. Clin Exp Hypertens.

[28] Rhee MY, Baek SH, Kim W, Park CG, Park SW, Oh BH (2015). Efficacy of fimasartan/hydrochlorothiazide combination in hypertensive patients inadequately controlled by fimasartan monotherapy. Drug Des Devel Ther.

[29] Sachse A, Verboom CN, Jäger B (2002). Efficacy of eprosartan in combination with HCTZ in patients with essential hypertension. J Hum Hypertens.

[30] Julius S, Kjeldsen SE, Weber M, Brunner HR, Ekman S, Hansson L (2004). VALUE trial group, outcomes in hypertensive patients at high cardiovascular risk treated with regimens based on valsartan or amlodipine: the VALUE randomised trial. Lancet..

[31] Higgins JP, Thompson SG, Deeks JJ, Altman DG (2003). Measuring inconsistency in meta-analyses. BMJ..

[32] Mackay JH, Arcuri KE, Goldberg AI, Snapinn SM, Sweet CS (1996). Losartan and low-dose hydrochlorothiazide in patients with essential hypertension. Arch Intern Med.

[33] Sica D, Bakris GL, White WB, Weber MA, Cushman WC, Huang P (2012). Blood pressure-lowering efficacy of the fixed-dose combination of azilsartan medoxomil and chlorthalidone: a factorial study. J Clin Hypertens.

[34] Cushman WC, Bakris GL, White WB, Weber MA, Sica D, Roberts A (2012). Azilsartan medoxomil plus chlorthalidone reduces blood pressure more effectively than olmesartan plus hydrochlorothiazide in stage 2 systolic hypertension. Hypertension..

[35] Cushman WC, Bakris GL, White WB, Weber MA, Sica D, Roberts A (2018). A randomized titrate-to-target study comparing fixed-dose combinations of azilsartanmedoxomil and chlorthalidone with olmesartan and hydrochlorothiazide in stage-2 systolic hypertension. J Hypertens.

[36] Neutel JM, Cushman WC, Lloyd E, Barger B, Handley A (2017). Comparison of long-term safety of fixed-dose combinations azilsartan medoxomil/chlorthalidone vs olmesartan medoxomil/hydrochlorothiazide. J Clin Hypertens.

[37] Gaciong Z, Siński M, Lewandowski J (2013). Blood pressure control and primary prevention of stroke: summary of the recent clinical trial data and meta-analyses. Curr Hypertens Rep.

[38] Makai P, IntHout J, Deinum J, Jenniskens K, Wilt GJV (2017). A network meta-analysis of clinical management strategies for treatment-resistant hypertension: making optimal use of the evidence. J Gen Intern Med.

[39] Taylor AA, Shoheiber O (2003). Adherence to antihypertensive therapy with fixed-dose amlodipine besylate/benazepril HCl versus comparable component-based therapy. Congest Heart Fail.

[40] Taylor AA, Siragy H, Nesbitt S (2011). Angiotensin receptor blockers: pharmacology, efficacy, and safety. J Clin Hypertens (Greenwich).

[41] Cushman WC, Bakris GL, White WB, Weber MA, Sica D, Roberts A (2011). Efficacy and safety of azilsartan medoximil/chlorthalidone vs olmesartan/HCTZ combinations in stage 2 systolic hypertension. J Clin Hypertens (Greenwich).

[42] National Guideline Centre (UK) (2019). Hypertension in adults: diagnosis and management.

[43] Mukete BN, Ferdinand KC (2016). Polypharmacy in older adults with hypertension: a comprehensive review. J Clin Hypertens (Greenwich).

[44] Stuhec M, Gorenc K, Zelko E (2019). Evaluation of a collaborative care approach between general practitioners and clinical pharmacists in primary care community settings in elderly patients on polypharmacy in Slovenia: a cohort retrospective study reveals positive evidence for implementation. BMC Health Serv Res.

[45] Štuhec M, Potočin I, Stepan D, Ušaj L, Petek Šter M (2019). Potential drug interactions with antibacterials in long-term care facilities analyzed by two interaction checkers. Int J Clin Pharm.

[46] Hess NC, Carlson DJ, Inder JD, Jesulola E, McFarlane JR, Smart NA (2016). Clinically meaningful blood pressure reductions with low intensity isometric handgrip exercise. A randomized trial. Physiol Res.

[47] Chrysant SG, Chrysant GS (2004). Antihypertensive efficacy of olmesartan medoxomil alone and in combination with hydrochlorothiazide. Expert Opin Pharmacother.

[48] Chrysant SG (2003). Fixed combination therapy of hypertension: focus on valsartan/hydrochlorothiazide combination (Diovan/HCT). Expert Rev Cardiovasc Ther.

[49] Lacourcière Y (2002). A new fixed-dose combination for added blood pressure control: telmisartan plus hydrochlorothiazide. J Int Med Res.

[50] Maillard M, Burnier M (2005). Telmisartan/hydrochlorothiazide: a new fixed dose combination. Expert Rev Cardiovasc Ther.

[51] Melian EB, Jarvis B (2002). Candesartan cilexetil plus hydrochlorothiazide combination: a review of its use in hypertension. Drugs..

[52] Ram CV (2004). Antihypertensive efficacy of angiotensin receptor blockers in combination with hydrochlorothiazide: a review of the factorial-design studies. J Clin Hypertens (Greenwich).

[53] Multiple Risk Factor Intervention Trial Research Group (1990). Mortality after 10 1/2 years for hypertensive participants in the multiple risk factor intervention trial. Circulation.

[54] Dorsch M, Gillespie B, Erikson S (2011). Chlorthalidone reduces cardiovascular events compared with hydrochlorothiazide: a retrospective cohort analysis. Hypertension.

[55] Dhalla IA, Gomes T, Yao Z, Nagge J, Persaud N, Hellings C (2013). Chlorthalidone versus hydrochlorothiazide for the treatment of hypertension in older adults: a population-based cohort study. Ann Intern Med.

[56] Ernst M, Carter B, Goerdt C (2006). Comparative antihypertensive effects of hydrochlorothiazide and chlorthalidone on ambulatory and office blood pressure. Hypertension.

[57] Pareek AK, Messerli FH, Chandurkar NB, Dharmadhikari SK, Godbole AV, Kshirsagar PP (2016). Efficacy of lowdose chlorthalidone and hydrochlorothiazide as assessed by 24-h ambulatory blood pressure monitoring. JACC.

[58] Peterzan MA, Hardy R, Chaturvedi N, Hughes AD (2012). Meta-analysis of dose response relationships for hydrochlorothiazide, chlorthalidone, and bendroflumethiazide on blood pressure, serum potassium, and urate. Hypertension.

