# Supplementary material for: Combining angiotensin receptor blockers with chlorthalidone or hydrochlorothiazide – which is the better alternative? A meta-analysis
Source: Syst Rev. 2020 Aug 24;9:195. doi: 10.1186/s13643-020-01457-9 (PMC7445912; doi:10.1186/s13643-020-01457-9)
Supplement: Supplementary file 1 — Additional file 1: Table S1. Quality assessment results. Figure S1. Funnel plot for systolic blood pressure ARB/HCTZ versus ARB. Figure S2. Funnel plot for diastolic blood pressure ARB/HCTZ versus ARB. Figure S3. Funnel plot for systolic blood pressure ARB/CTLD vs ARB/HCTZ. Figure S4. Funnel plot for diastolic blood pressure ARB/CTLD vs ARB/HCTZ. Table S2. Sensitivity analysis SBP and DBP ARB/HCTZ vs ARB. Table S3. Sensitivity analysis SBP and DBP ARB/CTLD vs ARB/HCTZ. Table S4. Quality of the evidence according to the GRADE methodology for the 2 outcomes. [file 13643_2020_1457_MOESM1_ESM.docx]

**Table: S1 Quality assessment results**

| **Study** | **Selection bias** | **Study design** | **Confounders** | **Blinding** | **Data collection methods** | **Withdrawals and drop-outs** | **Global rating** |
| --- | --- | --- | --- | --- | --- | --- | --- |
| Benz et al, 1998 | strong | strong | strong | strong | strong | strong | **Strong** |
| Cushman et al, 2012 | strong | strong | strong | strong | strong | strong | **Strong** |
| Cushman et al, 2018 | strong | strong | strong | strong | strong | strong | **Strong** |
| Edes, 2009 | strong | strong | strong | strong | strong | strong | **Strong** |
| Fogari et al, 2010 | strong | strong | strong | strong | strong | strong | **Strong** |
| Kwon et al, 2013 | moderate | strong | strong | moderate | strong | strong | **Weak** |
| Lacourciere et al, 2001 | strong | strong | strong | strong | strong | strong | **Strong** |
| Lacourciere et al, 2005 | strong | strong | strong | strong | strong | moderate | **Moderate** |
| Lacourciere and Martin, 2002 | strong | strong | strong | strong | strong | moderate | **Moderate** |
| MacKay et al, 1996 | strong | strong | strong | strong | strong | strong | **Strong** |
| Makita et al, 2009 | moderate | strong | strong | moderate | strong | strong | **Weak** |
| Neutel et al, 2017 | strong | strong | strong | moderate | strong | strong | **Moderate** |
| Rhee et al, 2015 | strong | strong | moderate | strong | strong | strong | **Moderate** |
| Sachse et al, 2002 | strong | strong | strong | strong | moderate | strong | **Moderate** |
| Sica et al, 2012 | strong | strong | strong | strong | strong | strong | **Strong** |


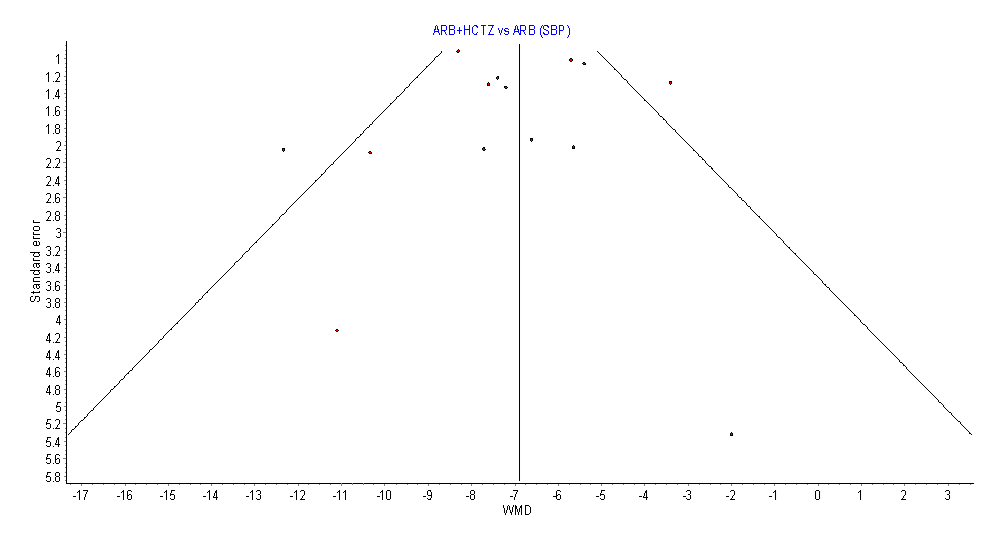


**Figure: S1 Funnel plot for systolic blood pressure ARB/HCTZ versus ARB**


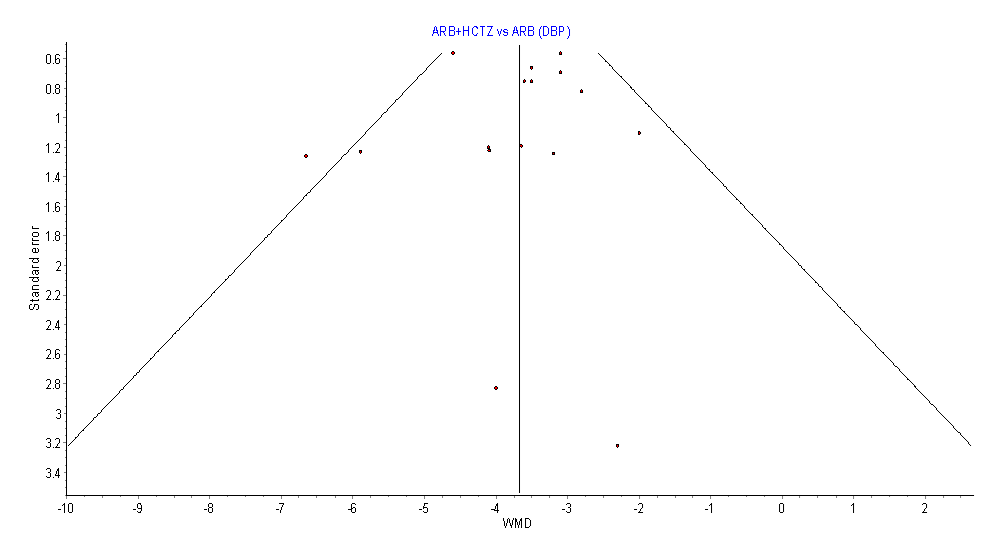


**Figure: S2 Funnel plot for diastolic blood pressure ARB/HCTZ versus ARB**


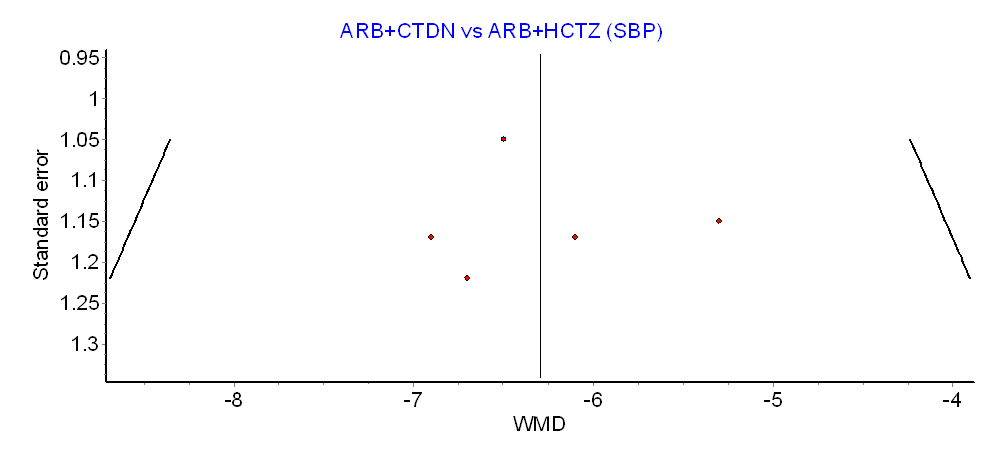


**Figure: S3. Funnel plot for systolic blood pressure ARB/CTLD vs ARB/HCTZ**


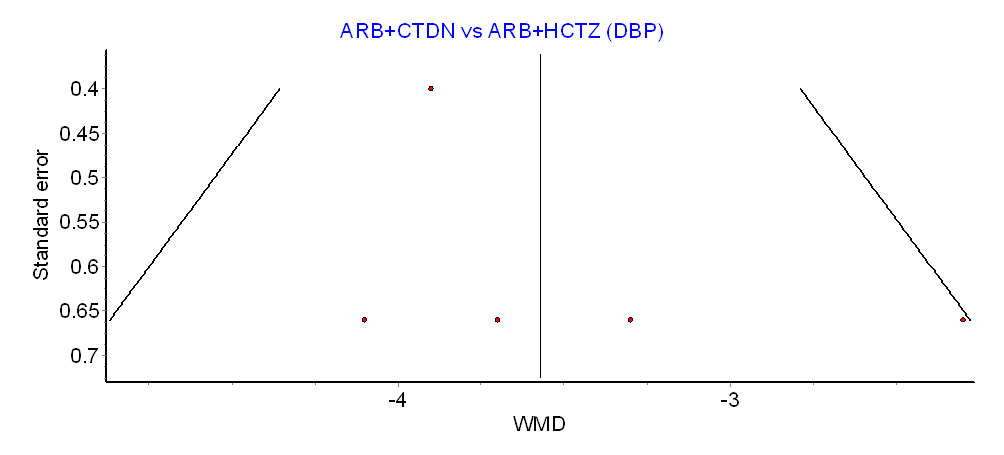


**Figure: S4. Funnel plot for diastolic blood pressure ARB/CTLD vs ARB/HCTZ**

**Table: S2 Sensitivity analysis SBP and DBP ARB/HCTZ vs ARB**

| **Excluded study** | **Systolic blood pressure ARB/HCTZ vs ARB** | | | | **Diastolic blood pressure ARB/HCTZ vs ARB** | | | |
| --- | --- | --- | --- | --- | --- | --- | --- | --- |
|  | **Pooled WMD** | **Cochran Q** | **p** | **I^2^** | **Pooled WMD** | **Cochran Q** | **p** | **I^2^** |
| Fogari et al (2010) [20] | -7.11 (-8.40, -5.82) | 23.65 | 0.0227 | 49.25 | -3.73 (-4.25, -3.21) | 16.72 | 0.2713 | 16.28 |
| Benz et al (1998) [21] | -6.77 (-7.95, -5.58) | 23.06 | 0.0272 | 47.97 | -3.60 (-4.03, -3.16) | 14.11 | 0.4417 | 0.76 |
| Benz et al (1998) [21] | -6.93 (-8.20, -5.67) | 25.52 | 0.0125 | 52.98 | -3.65 (-4.17, -3.14) | 17.35 | 0.2379 | 19.32 |
| Benz et al (1998) [21] | -6.69 (-7.73, -5.65) | 18.59 | 0.0990 | 35.44 | -3.58 (-4.01, -3.14) | 11.70 | 0.6302 | 0.00 |
| Benz et al (1998) [21] | -6.86 (-8.11, -5.59) | 25.75 | 0.0117 | 53.39 | -3.68 (-4.19, -3.17) | 17.32 | 0.2394 | 19.18 |
| Edes (2009) [22] | -6.59 (-7.87, -5.31) | 23.06 | 0.0272 | 47.97 | -3.50 (-3.97, -3.03) | 14.21 | 0.4342 | 1.48 |
| Kwon et al (2013) [23] | -6.91 (-8.12, -5.70) | 25.07 | 0.0145 | 52.13 | -3.67 (-4.17, -3.16) | 17.46 | 0.2324 | 19.83 |
| Lacourciere and Martin (2002) [24] | -6.83 (-8.17, -5.49) | 25.72 | 0.0118 | 53.34 | -3.69 (-4.23, -3.15) | 17.40 | 0.2353 | 19.55 |
| Lacourciere et al (2001) [25] | -7.08 (-8.40, -5.67) | 24.34 | 0.0183 | 50.69 | -3.77 (-4.29, 3.25) | 16.26 | 0.2978 | 13.90 |
| Lacourciere et al (2005) [26] | -6.86 (-8.19, -5.53) | 25.86 | 0.0112 | 53.59 | -3.67 (4.21, -3.14) | 17.47 | 0.2321 | 19.85 |
| Lacourciere et al (2005) [26] | -6.82 (-8.14, -5.50) | 25.59 | 0.0123 | 53.10 | -3.68 (-4.22, -3.15) | 17.42 | 0.2344 | 19.64 |
| MacKay et al (1996) [30] | - | - | - | - | -3.74 (-4.20, -3.27) | 15.08 | 0.3727 | 7.17 |
| MacKay et al (1996) [30] | - | - | - | - | -3.65 (-4.17, -3.14) | 17.34 | 0.2384 | 19.27 |
| Makita et al (2009) [27] | -6.85 (-8.06, -5.64) | 24.87 | 0.0155 | 51.74 | -3.67 (-4.18, -3.17) | 17.29 | 0.2408 | 19.05 |
| Rhee et al (2015) [28] | -6.90 (-8.18, -5.62) | 25.89 | 0.0111 | 53.66 | -3.67 (-4.18, -3.15) | 17.48 | 0.2317 | 19.89 |
| Sachse et al (2002) [29] | -7.23 (-8.28, -6.18) | 17.76 | 0.1233 | 32.42 | -3.74 (-4.24, -3.23) | 16.27 | 0.2973 | 13.94 |

**Table: S3 Sensitivity analysis SBP and DBP ARB/CTLD vs ARB/HCTZ**

| **Excluded study** | **Systolic blood pressure ARB/CTLD vs ARB/HCTZ** | | | | **Diastolic blood pressure ARB/CTLD vs ARB/HCTZ** | | | |
| --- | --- | --- | --- | --- | --- | --- | --- | --- |
|  | **Pooled WMD** | **Cochran Q** | **p** | **I^2^** | **Pooled WMD** | **Cochran Q** | **p** | **I^2^** |
| Cushaman et al (2012) [32] | -6.54 (-7.67, -5.42) | 0.25 | 0.9683 | 0.00 | -3.80 (-4.34, -3.25) | 0.87 | 0.8337 | 0.00 |
| Cushaman et al (2012) [32] | -6.15 (-7.27, -5.03) | 0.86 | 0.8345 | 0.00 | -3.48 (-4.18, -2.78) | 4.48 | 0.2137 | 33.10 |
| Cushaman et al (2018) [33] | -6.34 (-7.46, -5.22) | 1.16 | 0.7633 | 0.00 | -3.62 (-4.38, -2.87) | 5.03 | 0.1694 | 40.40 |
| Cushaman et al (2018) [33] | -6.21 (-7.32, -5.10) | 1.06 | 0.7870 | 0.00 | -3.55 (-4.32, -2.78) | 5.19 | 0.1584 | 42.20 |
| Neutel et al (1997) [34] | -6.23 (-7.39, -5.08) | 1.14 | 0.7668 | 0.00 | -3.35 (-4.11, -2.59) | 4.11 | 0.2499 | 26.99 |

The following search strategy was applied: Search strategy in PUBMED

Period covered: until July 2020

Date of search: 30.07.2020

01. diuretics/(102164)

02. diuretics.tw (0)

03. hydrochlorothiazide/(9025)

04. hydrochlorothiazide.tw (9174)

05. HCTZ/ (9039)

06. HCTZ.tw (0)

07. chlorthalidone/ (1874)

08. chlorthalidone.tw (0)

09. CTLD/ (121)

10. CTLD.tw (0)

11. CTN/ (2294)

12. CTN.tw (0)

13. 01 or 02 or 03 or 04 or 05 or 06 or 07 or 08 or 09 or 10 or 11 or 12 (106047)

14. angiotensin receptor blocker/ (21173)

15. angiotensin receptor blocker.tw (50843)

16. ARB/ (6171)

17. ARB.tw (0)

18. 14 or 15 or 16 or 17 (50848)

19. 13 and 18 (5619)

20. hypertension/ (537442)

21. hypertension.tw (0)

22. blood pressure/ (615922)

23. blood pressure.tw (4904653)

24. 20 or 21 or 22 or 23 (5189084)

25. 19 and 24 (4095)

26. trial/ (1732480)

27. trial.tw (0)

28. clinical trial/ (1195486)

29. clinical trial.tw (5530905)

30. controlled/ (1289783)

31. controlled.tw (0)

32. randomi*/ (896886)

33. randomi*.tw (0)

34. blind/ (306454)

35. blind.tw (0)

36. double blind/ (197929)

37. double blind.tw (563695)

38. 26 or 27 or 28 or 29 or 30 or 31 or 32 or 33 or 34 or 35 or 36 or 37 (6787913)

39. 25 and 38 (2745)

We applied the same structure of the search strategy with the subset of Cyrillic keywords. Unfortunately, that subset did not turn any valuable results. Additionally, we did not use MESH terms as we did not want to limit the results to a subset of PubMed results as it would take up to several week for a new citation to be indexed.

**Table S4: Quality of the evidence according to the GRADE methodology for the 2 outcomes**

| **Outcome** | **Number and design of studies** | **Study limitations (Risk of bias)** | **Inconsistency** | **Indirectness** | **Imprecision** | **Publication bias** | **Upgrading factors** | **Quality of evidence** |
| --- | --- | --- | --- | --- | --- | --- | --- | --- |
| Changes of SBP | 14 RCT | No serious limitations | No serious inconsistency | Serious (-1) | No serious imprecision | Undetected | No upgrades | Moderate |
| Changes of DBP | 14 RCT | No serious limitations | No serious inconsistency | Serious (-1) | No serious imprecision | Undetected | No upgrades | Moderate |
